# Correlation between the Level of Social Distancing and Activity of Influenza Epidemic or COVID-19 Pandemic: A Subway Use-Based Assessment

**DOI:** 10.3390/jcm10153369

**Published:** 2021-07-29

**Authors:** Hye Seong, Jin-Wook Hong, Hak-Jun Hyun, Jin-Gu Yoon, Ji-Yun Noh, Hee-Jin Cheong, Woo-Joo Kim, Jae-Hun Jung, Joon-Young Song

**Affiliations:** 1 Department of Internal Medicine, Korea University College of Medicine, Guro Hospital, Seoul 08308, Korea; msmjoonhoo@gmail.com (H.S.); hak-neck@hanmail.net (H.-J.H.); zephirisj9@gmail.com (J.-G.Y.); jynoh@korea.ac.kr (J.-Y.N.); heejinmd@korea.ac.kr (H.-J.C.); wjkim@korea.ac.kr (W.-J.K.); 2 Artificial Intelligence and Big-Data Convergence Center, Gachon University College of Medicine and Science, Incheon 21565, Korea; awesomehjw@gmail.com; 3 Department of Preventive Medicine, Gachon University College of Medicine, Incheon 21565, Korea; 4 Asian Pacific Influenza Institute, Seoul 08308, Korea

**Keywords:** COVID-19, severe acute respiratory syndrome coronavirus 2, social distancing, subway, public health

## Abstract

Social distancing is an effective measure to mitigate the spread of novel viral infections in the absence of antiviral agents and insufficient vaccine supplies. Subway utilization density may reflect social activity and the degree of social distancing in the general population.; This study aimed to evaluate the correlations between subway use density and the activity of the influenza epidemic or coronavirus disease 2019 (COVID-19) pandemic using a time-series regression method. The subway use-based social distancing score (S-SDS) was calculated using the weekly ridership of 11 major subway stations. The temporal association of S-SDS with influenza-like illness (ILI) rates or the COVID-19 pandemic activity was analyzed using structural vector autoregressive modeling and the Granger causality (GC) test. During three influenza seasons (2017–2020), the time-series regression presented a significant causality from S-SDS to ILI (*p* = 0.0484). During the COVID-19 pandemic in January 2020, S-SDS had been suppressed at a level similar to or below the average of the previous four years. In contrast to the ILI rate, there was a negative correlation between COVID-19 activity and S-SDS. GC analysis revealed a negative causal relationship between COVID-19 and S-SDS (*p* = 0.0098).; S-SDS showed a significant time-series association with the ILI rate but not with COVID-19 activity. When public transportation use is sufficiently suppressed, additional social mobility restrictions are unlikely to significantly affect COVID-19 pandemic activity. It would be more important to strengthen universal mask-wearing and detailed public health measures focused on risk activities, particularly in enclosed spaces.

## 1. Introduction

The severe acute respiratory syndrome coronavirus 2 outbreak has spread worldwide in a short period of time after its first documented emergence in December 2019. The World Health Organization declared it a pandemic on 11 March 2020 [1]. During the early stages of the coronavirus disease (COVID-19) pandemic, each country conducted different public health interventions, which contributed to the disparities pandemic’s incidence and mortality [2,3].

In the absence of effective antiviral agents and sufficient vaccine supplies, nonpharmacologic interventions, especially social distancing, are the essential measures to mitigate epidemic viral infections, such as COVID-19 and novel influenza [4]. Social distancing measures include school closures, workplace measures/closures, and avoidance of crowding. The government can also ban public gatherings and restrict visits to public places, such as churches, theaters, and gymnastics, to avoid crowding. Since people often move to places of interest using public transportation, including subways and buses, it is usually congested, thereby raising concerns regarding COVID-19 transmission through public transportation [5]. In addition, the presence of a high-speed railway station was associated with the pandemic spread of COVID-19 according to the early studies conducted in China [6].

Because subway use density reflects the social activities including daily commuting, eating out, and personal meeting, it would be a proxy for social distancing. However, irrespective of subway use density, crowding could happen in bars, restaurants, sports events, and any places of interest. Thus, the level of social distancing can be influenced by various factors such as subway usage rate, visit rate to indoor places of interest, and weather (increase in indoor activities). Herein, we evaluated the association between subway use density and the activity of the influenza epidemic or COVID-19 pandemic using the time-series regression method. 

## 2. Materials and Methods

### 2.1. Study Design and Data Collection

In this study, we quantitatively measured social distancing levels using subway ridership for 11 major subway stations and assessed their time-series associations with influenza or COVID-19 pandemic activity.

For the influenza activity, weekly influenza-like illness (ILI) rates (cases/1000 patients) were estimated from week 41 of 2017 to week 20 of 2020, using data from the Korea Disease Control and Prevention Agency (KDCA) [7]. The number of weekly domestic COVID-19 cases was counted from week 5 of 2020 to week 5 of 2021 based on the KDCA reports [8]. We estimated the level of social distancing using the subway use-based social distancing score (S-SDS). The subway passenger ridership recorded by five regional metropolitan express transit corporations was collected to determine the S-SDS.

### 2.2. Subway Use-Based Social Distancing Score

**Figure 1:**
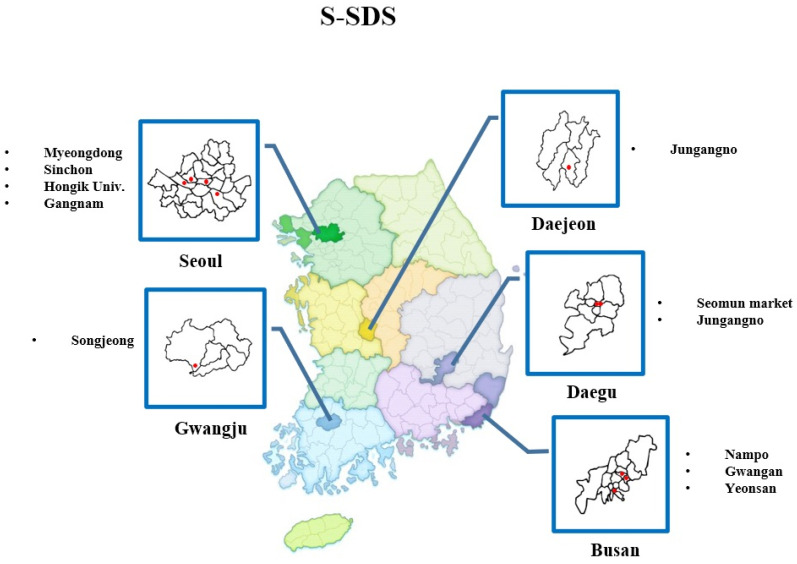
Major subway stations selected to estimate S-SDS. S-SDS was determined based on the number of passengers for 11 major subway stations in five cities of the Republic of Korea. S-SDS, subway use-based social distancing score.

From week 41 of 2017 to week 5 of 2021, we identified the ridership of 11 major subway stations in five cities (Seoul: 4, Daejeon: 1, Daegu: 2, Gwangju: 1, and Busan: 3) to calculate the weekly S-SDS (Figure 1) [9,10,11,12,13]. To calculate S-SDS, we first obtained the average number of weekly passengers for each station during the last four years. Thereafter, we divided the number of weekly subway passengers by the average weekly value to normalize the score, as shown in the following equation:

$$\mathrm{Subway use}-\mathrm{based SDS}=\frac{\mathrm{The number of passengers at the subway stations per week}}{\mathrm{The average number of weekly passengers in the last 4 years}}$$

### 2.3. Data Analysis

The levels of social distancing for influenza or COVID-19 were suggested and statistically analyzed using the vector autoregressive (VAR) model, which is a useful tool for the analysis of multivariate time series [14] and is especially used to describe the dynamic behavior of time series and forecasting. Unlike the general correlation function, the VAR model has applied autocorrelation as serial correlation, which correlates a signal with a lagged of itself as a function of lag. This means that the VAR model is instantaneous interaction between *Y* and *X* through the process at different times. For example, suppose both *Y* and *X* are endogenous; in that case, the regressor includes the current value of endogenous variables in the structural form. Furthermore, our VAR model does not need covariates due to describing only the dynamic relationships between two time-series as S-SDS and ILI or COVID-19 without forecasting. The detailed VAR model is as follows:

$$\left[\begin{array}{c} X_{1,t}\\ X_{2,t}\\ \begin{array}{c} \vdots \\ X_{N,t} \end{array} \end{array}\right]=\left[\begin{array}{c} C_{1}\\ C_{2}\\ \begin{array}{c} \vdots \\ C_{N} \end{array} \end{array}\right]+\underset{i=1}{\sum }p{\left(\begin{array}{c} \begin{array}{ccc} \varphi_{11} & \ldots & \varphi_{1N}\\ \vdots & \ddots & \vdots \end{array}\\ \begin{array}{ccc} \varphi_{N1} & \ldots & \varphi_{NN} \end{array} \end{array}\right)}^{i}\;\left[\begin{array}{c} X_{1,t-i}\\ X_{2,t-i}\\ \begin{array}{c} \vdots \\ X_{N,\;t-i} \end{array} \end{array}\right]+\left[\begin{array}{c} \varepsilon_{1,t}\\ \varepsilon_{2,t}\\ \begin{array}{c} \vdots \\ \varepsilon_{N,t} \end{array} \end{array}\right]$$

where *X*_1_, *X*_2_ are time-series variables, respectively.

The methodological framework of our study comprised three main steps. The first step, data pre-processing, was to control the effects of seasonality and non-stationarity in the ILI rates, which were found to be non-stationary with seasonality in the univariate time series analysis, using the autoregressive integrated moving average model. Using the unit root test, we controlled the ILI rate, S-SDS, and COVID-19 occurrence residuals to follow white noise as stationary in the VAR model and checked for autoregression terms such as AR. The second step was to identify causal relationships between S-SDS and ILI rates or COVID-19 occurrence, for which the Granger causality (GC) test was used. The third and final steps were model adaptations. The final VAR model selected the lowest Akaike information criterion value from the second step.

Data processing and statistical analyses were conducted using SAS version 9.4 (SAS Institute, Cary, NC, USA). Each statistical test performed was two-tailed, and a *p*-value of less than 0.05 was considered significant.

## 3. Results

### 3.1. Trends of ILI Rate, COVID-19 Pandemic, and S-SDS

From week 41 of 2017 to week 5 of 2021, on average, 60,628 passengers (range: 6270–204,144) used 11 subway stations in five cities per week. The average S-SDS was 1.82 ± 0.08, 1.83 ± 0.06, and 1.43 ± 0.41 during the flu seasons of 2017–2018, 2018–2019, and 2019–2020, respectively (*p* < 0.01). In week 52 of all three years, the S-SDS reached its summit (1.98 in 2017–2018; 1.99 in 2018–2019, and 2.02 in 2019–2020). Since the first documented emergence of COVID-19 in the Republic of Korea at week 5 of 2020, the weekly S-SDS decreased sharply and was maintained close to 1 (1.08 ± 0.19) for a year (Supplementary Tables S1 and S2).

**Figure 2:**
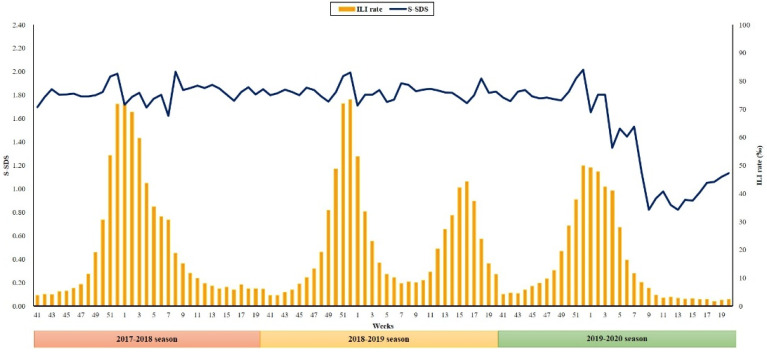
Weekly trend of ILI rates and S-SDS. The weekly trend of ILI rates and S-SDS during three consecutive flu seasons (2017–2018, 2018–2019 and 2019–2020 seasons) is shown. Weekly trend of ILI rate is shown as a bar graph and that of S-SDS is shown as a line graph. ILI, influenza-like illness; S-SDS, subway use-based social distancing score.

Supplementary Table S1 and Figure 2 present the weekly trends of ILI rates and S-SDS in the Republic of Korea during the 2017–2020 flu season (from week 41 in 2017 to week 20 in 2020). Across three consecutive study seasons, the ILI rates reached their peaks of 72.10, 73.30, and 49.80 during week 1 of 2017–2018, week 52 of 2018–2019, and week 52 of 2019–2020, respectively.

**Figure 3:**
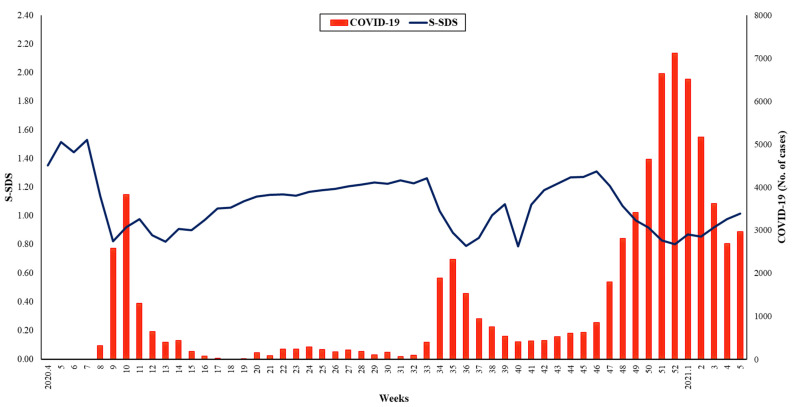
COVID-19 pandemic activity and S-SDS. COVID-19 cases were presented during study periods, between week 5 of 2020 and week 5 of 2021. The weekly trend of COVID-19 activity is shown as a bar graph and that of S-SDS is shown as a line graph. COVID-19, coronavirus disease 2019; S-SDS, subway use-based social distancing score.

As shown in Figure 3 and Supplementary Table S2, three waves passed during the first year of the COVID-19 pandemic in the Korea. The peaks of daily COVID-19 cases were at week 10 (*n* = 3832; first wave), week 35 (*n* = 2312; second wave), and week 52 (*n* = 7119; third wave) of 2020, respectively. Before the first and second waves, S-SDS was above 1.00, although it decreased below 1.00 with the start of each wave thereafter.

The S-SDS remained greater than 1.00 until two weeks before the first wave peak, although it decreased to less than 1.00 in the week before the peak, which continued until six weeks after the peak. During the second wave, the S-SDS exceeded 1.00 until one week before the peak, although it decreased to less than 1.00, which was maintained for another two weeks after the peak. During the third wave, the S-SDS was maintained below 1.00 from three weeks before the peak to four weeks after the peak.

### 3.2. Time-Series Associations between S-SDS and the Influenza Epidemic or COVID-19 Pandemic Activity

Using the VAR model, we investigated the time-series associations between S-SDS and the ILI rate or COVID-19 activity (number of cases). The time-series regression models for the degree of S-SDS and influenza activity during the three consecutive seasons are summarized in Table 1 and Supplementary Figure S1. While S-SDS had significantly positive correlations with the ILI rate reported two weeks (1.137%, *p* = 0.0242), three weeks (1.286%, *p* = 0.0107), and four weeks (0.613%, *p* = 0.0137) before, respectively, no inverse association was observed. The plots of the correlation functions and autocorrelation functions are shown in Supplementary Figures S3 and S4.

Table 1 also presents the VAR estimation results, which indicated that the COVID-19 pandemic presented a negative correlation with S-SDS calculated two weeks before (−28.37%, *p* = 0.0154), although there was no inverse correlation.

### 3.3. Causal Relationship between S-SDS and the ILI Rate or COVID-19 Activity

As presented in Table 2, the GC test results suggest that the increase in ILI rate was caused by a previous increase in S-SDS (*p* = 0.0484), although there was no inverse causality between the ILI rate and S-SDS (Table 2 and Supplementary Figures S2–S4).

GC tests at the level of COVID-19 activity and S-SDS were also performed, including the optimal lag orders. There was a consistent trend between COVID-19 activity and S-SDS. The results indicate that the COVID-19 outbreak influenced S-SDS (*p* = 0.0098), whereas a reverse causal relationship did not exist.

## 4. Discussion

This study showed that social distancing assessment based on subway use would be useful in predicting future influenza epidemics. Due to the COVID-19 pandemic, the S-SDS for the 2019‒2020 influenza season was much lower compared to the previous two seasons (mean S-SDS: 1.82 versus 1.83 versus 1.43 during the 2017–2018, 2018–2019, and 2019–2020 influenza seasons, respectively; *p* < 0.01). Accordingly, the average and peak ILI rates were the highest in the 2018–2019 season (23.20 and 73.30, respectively), followed by the 2017–2018 season (21.26 and 72.10, respectively) and 2019–2020 season (14.75 and 49.80, respectively). The ILI rates started to increase during the 2017–2018 and 2018–2019 seasons, when S-SDS crossed 1.8–1.9, although seasonal influenza epidemics were rapidly suppressed during the 2019–2020 season when S-SDS decreased below 1.4.

Interestingly, unlike influenza, S-SDS was not predictive of COVID-19 activity during the study period. The public has been concerned regarding the spread of COVID-19 through public transportation, although this study showed that the COVID-19 pandemic activity was not significantly affected by subway use density. This result is consistent with the findings of Severo et al. [15]. This can be explained by two aspects. First, the S-SDS during the COVID-19 pandemic might have been kept too low to influence COVID-19 activity due to its change. The average S-SDS value from Week 5 of 2020 to Week 5 of 2021 was significantly lower than the previous two years. In addition, the incidence of other respiratory virus infections during the COVID-19 pandemic was also significantly lower than the previous four-year mean cumulative incidence in the preintervention period (24.5%) in the Republic of Korea [16]. As a bundle of non-pharmaceutical public health interventions, mask-wearing, hand hygiene, contact tracing, and social distancing have been emphasized, social activity has decreased. Therefore, it may be difficult to predict the spread of novel infectious diseases through subway use-based social activities under intense non-pharmaceutical public health interventions. However, in the absence of solid non-pharmaceutical intervention, social activity through subway using may help predict the transmission of infectious diseases. Second, regarding the transmission mode of COVID-19, direct personal contacts (face-to-face talking and meal sharing) with previously confirmed cases were associated with disease transmission but not indirect contact in public transportation [17,18]. COVID-19 has been spreading through private gatherings rather than indirect contact in public transportation, making it challenging to predict COVID-19 pandemic activity. Moreover, COVID-19 can be easily transmitted via private meetings because of its unique properties, including asymptomatic transmission, insidious symptom onset, and high viral shedding during the early stages of infection [19].

While we found a probable link between public transportation and ILI rate and did not find a possible correlation between public transportation and COVID-19, several confounding factors could have influenced the results, including contact environment, climate (temperature and humidity), and herd immunity of population. There are three environmental settings where transmission of the SARS-CoV-2 spreads more easily: (1) Crowded places; (2) Close contact settings, especially where people have conservations very near each other; (3) Confined and enclosed spaces with poor ventilation.

Further analysis will be required across several seasons to verify whether there is a reliable cutoff value of S-SDS, which would be able to influence the activity of the influenza epidemic or COVID-19 pandemic. Depending on the transmissibility of each respiratory pathogen and herd immunity of the population, varying degrees of social distancing might be required to mitigate pandemic/epidemic activity. Compared with the seasonal influenza epidemic, which can be mitigated by vaccination, higher levels of social distancing may be required in a pandemic of novel respiratory diseases.

This study has some limitations. First, it was conducted in the Republic of Korea, a densely populated country. Depending on the population density and culture of each country, the level of social distancing required to curb or mitigate epidemics of respiratory virus infections may vary. Second, the study did not consider climatic factors and confounders previously mentioned, which are likely to influence viral activity and transmission, in addition to social distancing. Although we did not consider herd immunity, the COVID-19 vaccine was not available, and the SARS-CoV-2 seroprevalence was quite low (less than 0.07%) during the study period [20]. Finally, since this was a sort of ecological study, we could not view the influence of individual factors such as socioeconomic status and social activity levels. Although we found a probable link between public transportation and ILI spread, the data in this study might be influenced by confounding factors such as cold weather and holiday shopping in neighboring places, which needs to be controlled. Thus, it is important that future epidemiological and modeling studies consider these factors.

## 5. Conclusions

In conclusion, S-SDS showed a significant time-series association with the ILI rate but not with COVID-19 activity. When public transportation is below a certain threshold, additional social mobility restrictions do not affect COVID-19 pandemic activity significantly. The threshold might be variable geographically and temporally according to the population density, hygiene behavior, culture, climate, and diverse social factors. It would be more important to strengthen universal mask-wearing and detailed public health measures focused on risk activities, particularly in enclosed spaces. The findings of this study can support the transition from initial expert opinion-based practices to more evidence-based decision-making for public health interventions.

## Figures and Tables

**Table 1:** Dynamic relationship (vector autoregressive model) between subway use-based social distancing score and influenza-like illness/coronavirus disease 2019.

Indicator	Variable	Coefficient	Standard Error	*t*-Statistic	Probability *> |t|*
S-SDS and ILI rate					
S-SDS	ILI (t-1)	0.00362	0.00251	1.44	0.1519
	ILI (t-2)	0.01137	0.00497	2.29	0.0242
	ILI (t-3)	0.01286	0.00495	2.6	0.0107
	ILI (t-4)	0.00613	0.00244	2.51	0.0137
ILI	S-SDS (t-1)	3.11338	3.85642	0.81	0.4214
	S-SDS (t-2)	5.24173	4.69760	1.12	0.2672
	S-SDS (t-3)	1.08023	4.72556	−0.23	0.8197
	S-SDS (t-4)	6.71900	3.97062	1.69	0.0937
S-SDS and COVID-19 occurrence					
S-SDS	COVID-19 (t-1)	0	0.00003	−0.17	0.8690
	COVID-19 (t-2)	−0.00002	0.00004	−0.52	0.6081
	COVID-19 (t-3)	0.00001	0.00002	0.32	0.7475
COVID-19	S-SDS (t-1)	−1196.25	909.395	−1.32	0.1952
	S-SDS (t-2)	−2836.55	1125.70	−2.52	0.0154
	S-SDS (t-3)	−344.598	934.842	−0.37	0.7142

S-SDS, subway use-based social distancing score; ILI, influenza-like illness; COVID-19, coronavirus disease; ILI (t-1), ILI reported in a prior week; ILI (t-2), ILI reported in two weeks before; ILI (t-3), ILI reported in three weeks before; ILI (t-4), ILI reported in four weeks before; S-SDS (t-1), S-SDS calculated in a prior week; S-SDS (t-2), S-SDS calculated in two weeks before; S-SDS (t-3), S-SDS calculated in three weeks before; S-SDS (t-4), S-SDS calculated in four weeks before; COVID-19 (t-1), COVID-19 calculated in a prior week; COVID-19 (t-2), COVID-19 calculated in two weeks before; COVID-19 (t-3), COVID-19 calculated in three weeks before.

**Table 2:** Wald test for Granger causality between subway use-based social distancing score and influenza-like illness/coronavirus disease 2019.

Causality	df	Chi-Square	Probability > Chi-Square
S-SDS and ILI rate			
S-SDS → ILI	4	9.57	0.0484
ILI → S-SDS	4	8.96	0.0621
S-SDS and COVID-19 occurrence			
S-SDS → COVID-19	3	3.42	0.3311
COVID-19 → S-SDS	3	11.4	0.0098

S-SDS, subway use-based social distancing score; ILI, influenza-like illness; COVID-19, coronavirus disease 2019; df, degree of freedom.

## Data Availability

The data that support the findings of this study are available from the Korea Disease Control and Prevention Agency (KDCA) and the regional metropolitan express transit corporations. Data from KDCA are publicly available, the data from regional metropolitan express transit corporations were used under license for the current study. Data are however available from the authors upon reasonable request and with permission of the regional metropolitan express transit corporations.

## Supplementary Materials

The following are available online at https://www.mdpi.com/article/10.3390/jcm10153369/s1, Figure S1: Trend and correlation analysis and residual correlation diagnostics of ILI rate and S-SDS are shown. (a) Trend and correlation analysis for ILI rate, (b) Trend and correlation analysis for S-SDS, (c) Residual correlation diagnostics for ILI rate, (d) Residual correlation diagnostics for S-SDS, Figure S2: Trend and correlation analysis and residual correlation diagnostics of COVID-19 and SDS are shown. (a) Trend and correlation analysis for COVID-19, (b) Trend and correlation analysis for S-SDS, (c) Residual correlation diagnostics for COVID-19, (d) Residual correlation diagnostics for S-SDS, Figure S3: The plot of the correlation functions (CF). The plot of CF for (a) S-SDS and COVID-19; and (b) S-SDS and ILI rate. S-SDS, subway use-based social distancing score; COCID-19, coronavirus disease 2019; ILI, influenza-like illness. Figure S4: The plot of the autocorrelation functions (ACF) is presented. The plots of ACF for (a) S-SDS; (b) COVID-19; and (c) ILI rate. S-SDS, subway use-based social distancing score; COCID-19, coronavirus disease 2019; ILI, influenza-like illness. Table S1: Weekly trend of Subway use-based social distancing score (S-SDS), influenza-like illness (ILI) during three flu seasons. Table S2: Weekly trend of Subway use-based social distancing score (S-SDS) and COVID-19 occurrence between weeks 5 of 2020 and 5 of 2021.
