# Supplementary material for: Correlation between the Level of Social Distancing and Activity of Influenza Epidemic or COVID-19 Pandemic: A Subway Use-Based Assessment
Source: J Clin Med. 2021 Jul 29;10(15):3369. doi: 10.3390/jcm10153369 (PMC8348601; doi:10.3390/jcm10153369)
Supplement: Supplementary file 1 [file jcm-10-03369-s001.zip › jcm-1289457-supplementary.pdf]

Journal of
Clinical Medicine

Article

Supplementary Material: Correlation between the Level of Social Distancing and Activity of Influenza Epidemic or COVID-19 Pandemic: A Subway Use-Based Assessment

Hye Seong, Jin-Wook Hong, Hak-Jun Hyun, Jin-Gu Yoon, Ji-Yun Noh, Hee-Jin Cheong, Woo-Joo Kim, Jae-Hun Jung and Joon-Young Song

Table S1. Weekly trend of Subway use-based social distancing score (S-SDS), influenza-like illness (ILI) during three flu seasons.

**2017–2018 Season**																																
**Week**	**41**	**42**	**43**	**44**	**45**	**46**	**47**	**48**	**49**	**50**	**51**	**52**	**1**	**2**	**3**	**4**	**5**	**6**	**7**	**8**	**9**	**10**	**11**	**12**	**13**	**14**	**15**	**16**	**17**	**18**	**19**	**20**
S-SDS	1.70	1.78	1.85	1.80	1.81	1.81	1.79	1.79	1.80	1.82	1.96	1.98	1.72	1.78	1.82	1.70	1.77	1.80	1.62	2.00	1.84	1.86	1.88	1.86	1.89	1.86	1.80	1.75	1.83	1.87	1.81	1.85
ILI	3.80	4.20	4.10	5.20	5.30	6.30	7.70	11.40	19.00	30.70	53.60	71.80	72.10	69.00	59.60	43.60	35.30	31.70	30.70	18.80	15.10	11.70	9.80	8.00	7.20	6.20	6.80	5.80	7.60	6.20	6.10	6.00
**2018–2019 Season**																																
**Week**	**41**	**42**	**43**	**44**	**45**	**46**	**47**	**48**	**49**	**50**	**51**	**52**	**1**	**2**	**3**	**4**	**5**	**6**	**7**	**8**	**9**	**10**	**11**	**12**	**13**	**14**	**15**	**16**	**17**	**18**	**19**	**20**
S-SDS	1.80	1.82	1.85	1.83	1.80	1.86	1.84	1.79	1.74	1.83	1.96	1.99	1.71	1.80	1.80	1.84	1.74	1.76	1.90	1.89	1.83	1.85	1.85	1.84	1.82	1.82	1.78	1.73	1.80	1.94	1.82	1.83
ILI	3.70	3.80	4.90	5.70	7.80	10.10	13.20	19.20	34.00	48.70	71.90	73.30	53.10	33.60	23.00	15.30	11.30	10.20	8.00	8.60	8.30	9.10	12.10	20.30	27.20	32.20	42.10	44.20	37.30	23.90	15.10	11.30
**2019–2020 Season**																																
**Week**	**41**	**42**	**43**	**44**	**45**	**46**	**47**	**48**	**49**	**50**	**51**	**52**	**1**	**2**	**3**	**4**	**5**	**6**	**7**	**8**	**9**	**10**	**11**	**12**	**13**	**14**	**15**	**16**	**17**	**18**	**19**	**20**
S-SDS	1.78	1.75	1.83	1.84	1.79	1.77	1.78	1.77	1.76	1.83	1.94	2.02	1.65	1.80	1.80	1.35	1.51	1.45	1.53	1.14	0.82	0.92	0.98	0.86	0.82	0.91	0.90	0.97	1.05	1.06	1.10	1.14
ILI	4.20	4.6	4.50	5.80	7.00	8.20	9.70	12.70	19.50	28.50	37.80	49.80	49.10	47.80	42.40	40.90	28.00	16.40	11.60	8.50	6.30	3.90	2.90	3.20	2.80	2.50	2.70	2.30	2.40	1.70	2.10	2.30

S-SDS, subway use-based social distancing score; ILI, influenza-like illness.

Table S2. Weekly trend of Subway use-based social distancing score (S-SDS) and COVID-19 occurrence between weeks 5 of 2020 and 5 of 2021.

**2020 year**																											
**Week**	**5**	**6**	**7**	**8**	**9**	**10**	**11**	**12**	**13**	**14**	**15**	**16**	**17**	**18**	**19**	**20**	**21**	**22**	**23**	**24**	**25**	**26**	**27**	**28**	**29**	**30**	
S-SDS	1.51	1.45	1.53	1.14	0.82	0.92	0.98	0.86	0.82	0.91	0.90	0.97	1.05	1.06	1.10	1.14	1.15	1.15	1.14	1.17	1.18	1.19	1.21	1.22	1.23	1.22	
COVID-19	5	5	2	315	2578	3832	1301	639	395	437	187	76	32	11	22	156	84	233	237	291	226	179	217	187	111	168	
**2020–2021 year**																											
**Week**	**31**	**32**	**33**	**34**	**35**	**36**	**37**	**38**	**39**	**40**	**41**	**42**	**43**	**44**	**45**	**46**	**47**	**48**	**49**	**50**	**51**	**52**	**1**	**2**	**3**	**4**	**5**
S-SDS	1.25	1.23	1.26	1.03	0.88	0.79	0.85	1.00	1.08	0.79	1.08	1.18	1.22	1.27	1.27	1.31	1.21	1.07	0.97	0.92	0.83	0.80	0.87	0.86	0.92	0.98	1.01
COVID-19	69	101	392	1879	2317	1531	944	753	530	401	430	435	527	607	621	857	1790	2804	3415	4654	6644	7119	6519	5168	3621	2687	2969

S-SDS, subway use-based social distancing score.

Journal of
Clinical Medicine

MDPI

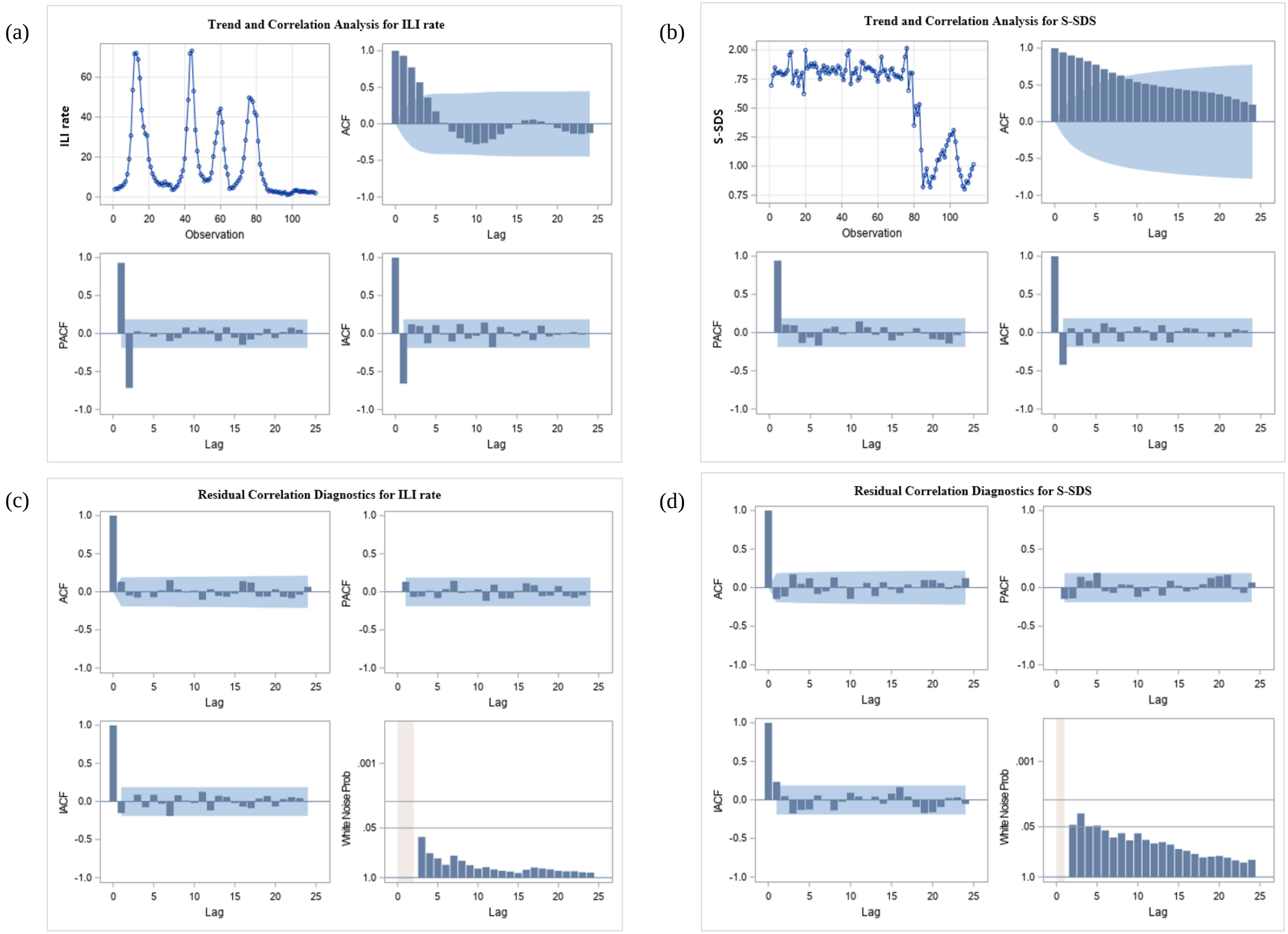

Figure S1. Trend and correlation analysis and residual correlation diagnostics of ILI rate and S-SDS are shown. (**a**) Trend and correlation analysis for ILI rate, (**b**) Trend and correlation analysis for S-SDS, (**c**) Residual correlation diagnostics for ILI rate, (**d**) Residual correlation diagnostics for S-SDS. ILI, influenza-like illness; S-SDS, subway use-based social distancing score.

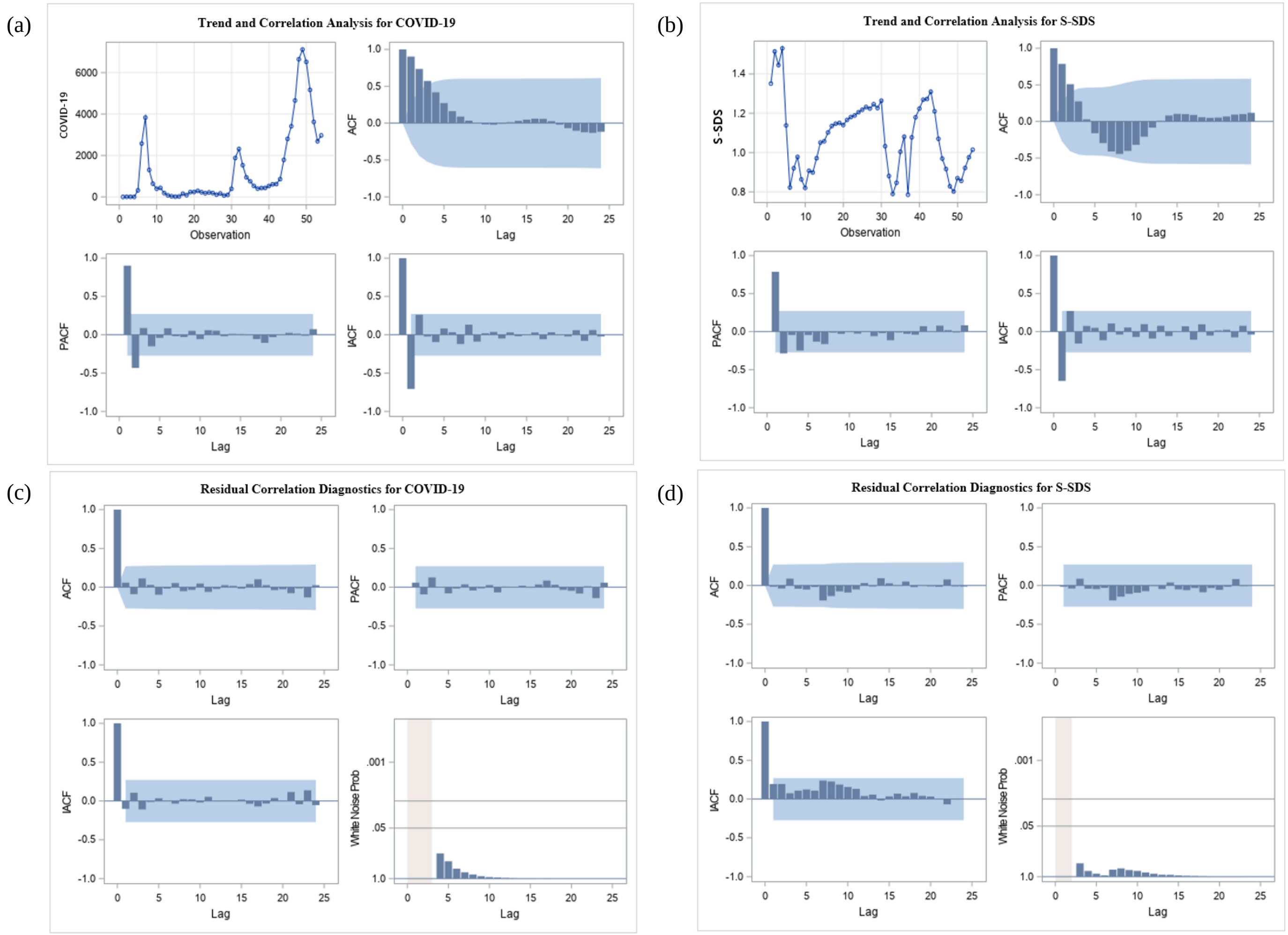

Figure S2. Trend and correlation analysis and residual correlation diagnostics of COVID-19 and SDS are shown. (**a**) Trend and correlation analysis for COVID-19, (**b**) Trend and correlation analysis for S-SDS, (**c**) Residual correlation diagnostics for COVID-19, (**d**) Residual correlation diagnostics for S-SDS. ILI, influenza-like illness; S-SDS, subway use-based social distancing score.

(a) CF (S-SDS, COVID-19)

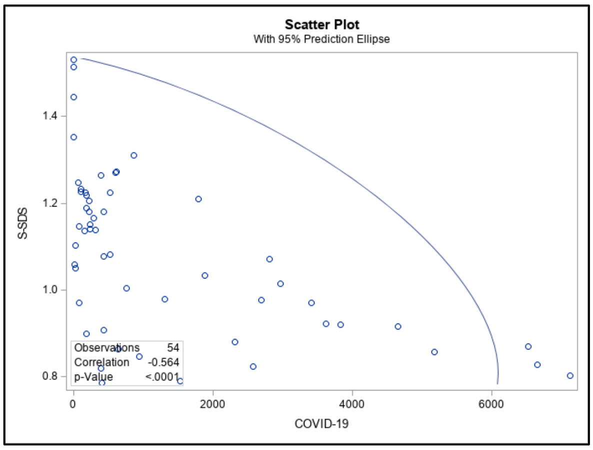

Coefficient: −0.56416 (p-value < 0.0001)

(b) CF (S-SDS, ILI rate)

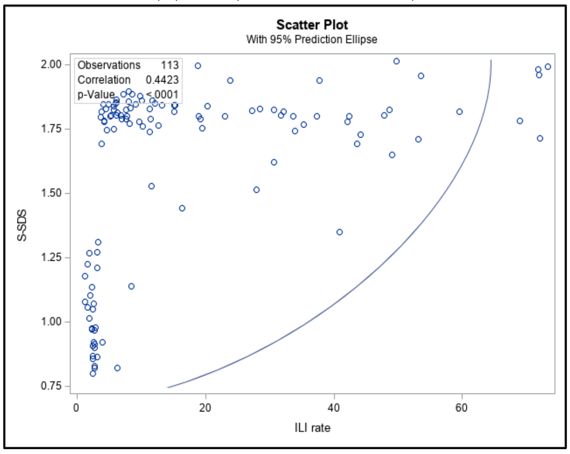

Coefficient: 0.44226 (p-value < 0.0001)

Figure S3. The plot of the correlation functions (CF). The plot of CF for (**a**) S-SDS and COVID-19; and (**b**) S-SDS and ILI rate. S-SDS, subway use-based social distancing score; COCID-19, coronavirus disease 2019; ILI, influenza-like illness.

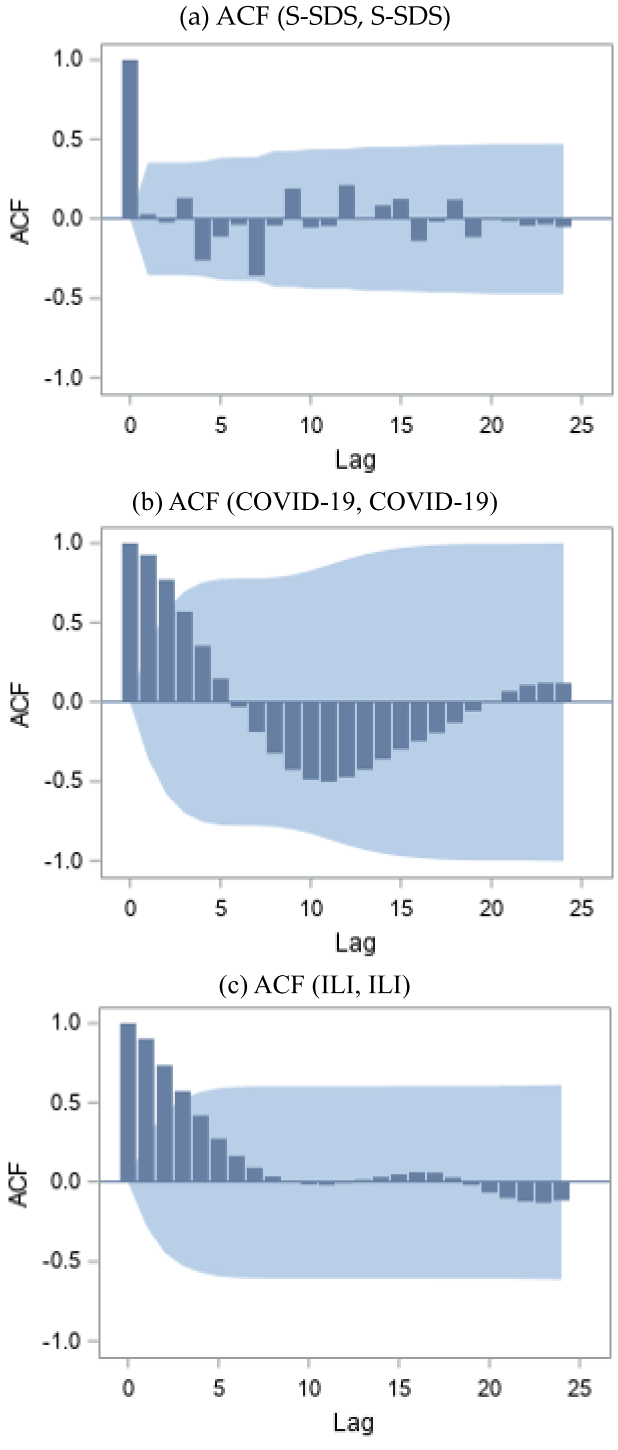

Figure S4. The plot of the autocorrelation functions (ACF) is presented. The plots of ACF for (**a**) S-SDS; (**b**) COVID-19; and (**c**) ILI rate. S-SDS, subway use-based social distancing score; COCID-19, coronavirus disease 2019; ILI, influenza-like illness.
